# Bacteria boost host NAD metabolism

**DOI:** 10.18632/aging.104219

**Published:** 2020-12-14

**Authors:** Igor Shats, Xiaoling Li

**Affiliations:** 1 Signal Transduction Laboratory, National Institute of Environmental Health Sciences, Research Triangle Park, NC 27709, USA

**Keywords:** host-microbe interaction, deamidated NAD synthesis, NAMPT inhibitors, bacterial nicotinamidase, nicotinamide riboside

Nicotinamide adenine dinucleotide (NAD) is an essential cofactor in multiple redox reactions. It is also consumed by DNA repair enzymes poly-(adenosine diphosphate–ribose) polymerases (PARPs), protein deacylases sirtuins, and cyclic ADP ribose hydrolase CD38 to regulate many cellular processes, including energy metabolism, genome stability, and immune responses. Mammalian cells have multiple sources for NAD synthesis, including tryptophan (*de novo* pathway), nicotinic acid (NA, deamidated salvage pathway), and the ribosylated intermediates such as nicotinamide riboside (NR) and nicotinamide mononucleotide (NMN). However, the main route of NAD biosynthesis in most mammalian cells and tissues is believed to be the amidated salvage pathway, where nicotinamide (NAM) is converted to NAD by a two-step pathway with nicotinamide phosphoribosyl transferase (NAMPT) as the rate limiting enzyme. The steady-state levels of NAD are tightly balanced by consumption and biosynthesis, and deregulation of this homeostasis is associated with decreased cellular NAD levels in aging. Consequently, various therapeutic strategies for elevating NAD levels have been proposed, including supplementation with NAD precursors such as NAM, NR and NMN, activation of NAMPT, or inhibition of CD38, (reviewed in [1]). Conversely, since rapidly proliferating cancer cells have elevated metabolic demands, inhibition of NAD metabolism has been considered as a potential antineoplastic therapeutic strategy. Since NAMPT-mediated amidated biosynthesis is believed to be the main NAD biosynthesis pathway, these development efforts have largely been focused on NAMPT inhibitors [2].

Whereas various cell-autonomous regulatory mechanisms have been described, the impact of microorganisms on mammalian NAD homeostasis remains largely unknown. In our recent study, we discovered that bacteria contribute to mammalian host NAD metabolism [3]. We found that different bacteria, including mycoplasma and *E.coli*, confer NAMPT inhibitors resistance to mammalian cells. Mechanistic investigation revealed that a microbial nicotinamidase (PncA) that converts nicotinamide to nicotinic acid, a key precursor in the alternative deamidated NAD salvage pathway, is necessary and sufficient for this protective effect. Particularly, we showed that intratumor bacteria confer resistance to a NAMPT inhibitor in a xenograft model [3]. These results suggest that the deamidated NAD biosynthesis pathway plays a more important role in tumor metabolism than previously recognized. Indeed, in a recent large survey of tumors and cancer cell lines from different cancer types, a large proportion of tumors have been demonstrated to contain genetic amplifications of the key enzymes (NAPRT and NADSYN) in the deamidated NAD biosynthesis pathway rendering these tumors exclusively dependent on the deamidated NAD biosynthesis [4].

Our further comparison of tissue distribution of NAD pathway metabolites from orally gavaged isotope-labelled NAD-boosting precursors, such as NAM and NR, in microbiota-depleted and regular mice revealed that these oral amidated supplements are primarily incorporated into tissue NAD through bacteria-enabled deamidated NAD salvage pathway. The bulk of NAM was deamidated in the GI tract of regular but not microbiota-depleted mice, and NAD boost in the liver was almost completely abolished in mice lacking gut microbiota. NR was partially deribosylated and converted to NAM even in the gut of microbiota-depleted mice and this process was further enhanced by bacteria. The resulting NAM was then deamidated to NA in a strictly microbiota-dependent manner. Microbiota-produced NA originating from both precursors was further metabolized by the host deamidated pathway to nicotinic acid adenine dinucleotide (NAAD), ultimately leading to the hepatic NAD boost. These isotope tracing experiments further showed that 85% of newly synthesized NAD in the liver and 65% in the kidney are produced via this microbiota-dependent deamidated pathway three hours after gavage of 80 mg/kg NAM, corresponding to the amount in commercial supplements. The deamidated pathway was also responsible for the bulk newly synthesized NAD in the liver after gavage of a lower 4 mg/kg dose of NAM, corresponding to steak meal for a human [3], highlighting the physiologic relevance of this mechanism. Furthermore, since liver-produced NAD is the main source of NAM that is secreted to circulation for NAD biosynthesis in all other tissues [5], our findings suggest that by enabling deamidated NAD synthesis in the liver, gut microbiota indirectly contribute to systemic NAD metabolism.

Our findings have a number of important implications. First, coupled with the results of Chowdhry et al. on the widespread dependence of tumors on the deamidated NAD synthesis [4], our results on the importance of bacteria in engaging the deamidated pathway suggest a potential role for antibiotics in cancer treatment by targeting NAD metabolism in patients with tumors addicted to the deamidated NAD biosynthesis. Along those lines, “anti-aging” amidated NAD boosting supplements, such as NAM, NR and NMN, should be taken with caution by cancer patients with tumors addicted to the deamidated NAD synthesis as these supplements might fuel tumor growth in a gut microbiota-dependent manner.

Secondly, our findings might provide a mechanistic basis for many gut dysbiosis associated pathologies, including aging. Our bioinformatic analysis suggests that pncA is an ubiquitous enzyme encoded by hundreds of bacterial species. However, although majority of species in some phyla, such as Bacteroidetes, encode putative pncA homologs, species from other phyla, such as Firmicutes, rarely do so. Thus, dysbiosis characterized by the increased Firmicutes/Bacteroidetes ratio, in the case of obesity, type 2 diabetes, and cardiovascular diseases [6], could potentially affect the total pncA activity of the microbiome, consequently deregulating the host NAD metabolism. Therefore, supplementation of pncA-expressing probiotics may help to alleviate these dysbiosis-associated pathologies.

Finally, our findings suggest that a combination of amidated NAD precursors with pncA-expressing probiotics might improve the efficiency of these supplements, while minimizing the undesirable side effects such as flushing associated with direct supplementation of NA.
